# Attentional control moderates the relationship between pain catastrophizing and selective attention to pain faces on the antisaccade task

**DOI:** 10.1038/s41598-020-69910-2

**Published:** 2020-07-30

**Authors:** Seyran Ranjbar, Mahdi Mazidi, Louise Sharpe, Mohsen Dehghani, Ali Khatibi

**Affiliations:** 1 Psychology Department, Shahid Beheshti University, Tehran, Iran; 2 Centre for the Advancement of Research on Emotion, The University of Western Australia, Crawley, WA Australia; 3 School of Psychology, The University of Sydney, Sydney, NSW Australia; 4 Centre of Precision Rehabilitation for Spinal Pain (CPR Spine), School of Sport, Exercise and Rehabilitation Sciences, College of Life and Environmental Sciences, University of Birmingham, Birmingham, B15 2TT UK; 5 Centre for Human Brain Health, University of Birmingham, Birmingham, UK

**Keywords:** Psychology, Cognitive neuroscience

## Abstract

Cognitive models of chronic pain emphasize the critical role of pain catastrophizing in attentional bias to pain-related stimuli. The aim of this study was (a) to investigate the relationship between pain catastrophizing and the ability to inhibit selective attention to pain-related faces (attentional bias); and (b) to determine whether attentional control moderated this relationship. One hundred and ten pain-free participants completed the anti-saccade task with dynamic facial expressions, specifically painful, angry, happy, and neutral facial expressions and questionnaires including a measure of pain catastrophizing. As predicted, participants with high pain catastrophizing had significantly higher error rates for antisaccade trials with pain faces relative to other facial expressions, indicating a difficulty disinhibiting attention towards painful faces. In moderation analyses, data showed that attentional control moderated the relationship between attentional bias to pain faces and pain catastrophizing. Post-hoc analyses demonstrated that it was shifting attention (not focusing) that accounted for this effect. Only for those with high self-reported ability to shift attention was there a significant relationship between catastrophizing and attentional bias to pain. These findings confirm that attentional control is necessary for an association between attentional bias and catastrophizing to be observed, which may explain the lack of relationships between attentional bias and individual characteristics, such as catastrophizing, in prior research.

## Introduction

Cognitive models of chronic pain propose pain catastrophizing as a risk factor that give rise to pain-related concerns and fuels attentional bias to pain-related information^1–6^. However, meta-analyses have failed to find relationships between theoretically important constructs, such as pain catastrophizing and attentional biases^7,8^. The absence of a relationship is problematic for these theories that suggest attentional biases are associated with pain catastrophizing^2–6^.

There are explanations for this lack of relationship. Todd et al.^1^ argued that attentional biases have a curvilinear relationship with threat and therefore relationships are obscured when assessed by simple correlations. Van Ryckegham et al.^9^ have argued that the context of attentional biases is important to whether biases towards or away from pain are helpful. Another possibility was raised by Dear et al.^10^, who found that the reliability of the dot-probe is poor, and reliability remains questionable for some indices using eye-tracking^11^. Another frequently raised issue is the lack of ecological validity of the stimuli (typically words). Numerous authors have argued that pain-related images^12^, facial expressions^13^ or somatosensory stimuli^5^ are more suitable to assess attentional biases in pain. Although research has used facial expression (e.g.^19^, all studies have used static faces, whereas Ceccarini and Caudek^14^, found that individuals were faster and more accurate in detecting an angry face in a crowd when the face images were dynamic.

It is also possible that the failure to observe predicted effects is due to the failure to identity moderators of the relationship between catastrophizing and attentional bias. One potential moderator is attentional control. Attentional control is defined as the effortful allocation of attention toward goal-relevant information in the face of conflicting prepotent attentional demands^15^. Heathcote et al.^16^ found that attentional control moderated the relationship between pain catastrophizing and attentional bias on a dot-probe task. They found a significant positive relationship between attentional control and vigilance towards pain faces only among healthy adolescents with high pain catastrophizing. In their second study^17^, they recorded eye movements of healthy children (aged 8–17) while looking at painful and neutral faces. They found a moderation effect of attentional control on the relationships between anxiety and attention to pain faces. Specifically, for children with low attentional control, higher anxiety was associated with a decreased dwell time on pain faces, whereas for those high in attentional control, the relationship was reversed. Lau et al.^18^ further investigated attentional control in adolescents by manipulating perceptual load and found that attentional biases for pain-related stimuli were observed in children with pain resulting in impairment compared with children without pain or those with low levels of impairment, but only under low perceptual loads where attentional control resources are available. Finally, Mazidi et al.^19^ found that attentional control moderated the relationship between pain catastrophizing and attention to happy faces in pain patients. Those with high attentional control and high pain catastrophizing focused more on happy faces (consistent with the vigilance-avoidance pattern identified).

To address limitations of previous research, we chose ecologically valid stimuli (dynamic painful facial expressions) and the anti-saccade task. The antisaccade task is a well-established assessment approach to examine individual differences in volitional control of attention^20^. The task starts with a central fixation cross followed by a single peripheral visual stimulus presented either on the left or the right side of the fixation cross. Participants are instructed to look either towards the stimulus (prosaccade trial) or away from the stimulus at the opposite side of the screen (antisaccade trial). Saccades in the prosaccade trials are considered a stimulus-driven, reflexive response. In contrast, antisaccades are more challenging than prosaccades. Antisaccade trials involves the processes of (a) inhibiting a prosaccade to the stimulus and (b) shifting attention to the opposite direction of the stimulus^21^. Participants’ error rates and reaction times are generally higher in antisaccade trials than prosaccade trials and the degree of slowing of responses in antisaccade trials compared with prosaccade trials is known as the antisaccade cost.

The degree to which errors and reaction times are greater in antisaccade trials is attributed to inhibitory attentional control^22,23^. This volitional control to inhibit a reflexive saccade to a stimulus in antisaccade trials has been shown to be modulated by the valence of emotional stimulus such as emotional facial expressions (e.g., see^24,25^. As such, the antisaccade task is capable of providing an index of attentional bias to emotional stimuli^26–28^. An index of attentional bias to emotional faces can be computed by subtracting the antisaccade cost (difference in reaction times for antisaccade versus prosaccade trials) of neutral stimuli from the antisaccade cost of emotional stimuli. The attention bias indicates the degree to which ability to engage in inhibitory attention is impaired by the presence of threatening stimuli^27,28^. There are other methods for measuring attention bias, such as the dot-probe paradigm^29^. Theoretically, attentional biases on the antisaccade task should be related to those on other measures, but on the antisaccade task the components of attention specifically affected are inhibition and attention switching^20^. One of the problems with the dot-probe task, based on reaction times, is that it cannot determine which aspects of attention are affected^30^.

In the present study, we hypothesized that participants with high pain catastrophizing would make more errors and take longer to attend away from painful (but not other) facial expressions than participants with low pain catastrophizing. We further hypothesized that attentional control would moderate the relationship between pain catastrophizing and attentional bias to pain faces.

## Material and methods

### Participants

Participants were volunteer undergraduate students recruited by advertisement from Shahid Beheshti University. They received either course credits or vouchers in exchange for their participation. The inclusion criteria were being at least 18 years old and having normal or corrected to normal vision. The exclusion criteria were a history of pain for at least three months or current pain, history of head or spinal trauma, neurological and psychiatric history or being under the influence of alcohol or other substances. One hundred and seventy-one individuals contacted the researcher to participate in the study. Of those, 18 participants were excluded due to current or previous pain problems, 19 were excluded due to frequent or current migraine or tension-type headaches and one individual reported a history of head injury. Five other individuals were excluded as they reported a history of mental health difficulties. The remaining 128 individuals were invited to take part in the experiment. There was a difficulty in calibration for 8 participants, and another participant left the session before completing all tasks. Nine participants were removed due to a technical problem in the recording of data during antisaccade trials. The final sample consisted of 110 participants (93 females). Participants were categorised into either high (n = 51) or low pain catastrophizing (n = 59) groups based on a median split of their scores on the Pain Catastrophizing Scale (PCS) (median score = 25). The Research was carried out according to the Helsinki declaration. The study was approved by the human research ethics committee of Shahid Beheshti University and all participants gave informed consent at the beginning of the session.

### Measures

#### Pain Catastrophizing Scale (PCS)^31^

The PCS is a 13-item self-report measure developed to assess individuals’ frequency of catastrophic thoughts relevant to painful experiences on 5-point Likert scale (0 = not at all to 4 = always). Higher scores indicate greater pain catastrophizing. The PCS has demonstrated adequate psychometric properties for both clinical and non-clinical Iranian samples^32,33^. Internal consistency (Cronbach’s alpha) in the present sample for the total score was 0.88.

#### Attentional control scale (ACS)^34^

The ACS is a self-report questionnaire that has been developed to measure individual differences in attentional control capacity. The scale contains 20 items that rated on a 4-point Likert scale ranging from (1 = almost never; 4 = always) with 11 items that are reverse-scored. Higher scores indicate greater self-reported ability to focusing and attentional shifting. The ACS has shown good adequate psychometric properties in an Iranian population^35^. In the current study, internal consistency (Cronbach’s alpha) was 0.8 for the total score and 0.79 and 0.86 for focusing and shifting subscales, respectively.

#### Fear of pain questionnaire-III (FPQ-III)^36^

The fear of pain questionnaire is a 30-item questionnaire that evaluates an individual’s fear of 30 painful incidents on a 5-point Likert scale ranging from (1 = not at all; 5 = extreme) with higher scores indicating greater fear of pain. The FPQ-III has shown good psychometric properties in both clinical and non-clinical populations^37^. The Persian version of the questionnaire has reported good psychometric properties in previous research^38^. In the current study, the Cronbach’s alpha was 0.92.

#### State-trait anxiety inventory (STAI)^39^

This inventory contains 40 items which measure the presence and severity of current symptoms of anxiety (state) and a generalized propensity to be anxious (trait). The participants are asked to complete the 20 items allocated to each of the state and trait subscales on a 4-point Likert scale. For the state items, the categories range from (1 = not at all) to (4 = very much), while for the trait items the range is from “1 = almost never” to “4 = almost always”. Higher scores indicate greater anxiety. In the current study, internal consistency (Cronbach’s alpha) was 0.93 and 0.91 for state and trait anxiety respectively.

#### The beck depression inventory-II (BDI-II)^40^

The BDI-II is a self-report questionnaire evaluating depressive symptoms over the past two weeks. It contains 21 items on a 4-point scale ranging from (0 = symptom absent) to (3 = severe). Higher scores indicate higher levels of depression. The Persian version of the BDI-II has shown robust psychometric properties^41^. The Cronbach’s alpha for the present study was 0.90.

### Apparatus and stimulus material

Eye movements were recorded using SensoMotoric Instruments (SMI) remote eye-tracker with 120 Hz sampling rate that uses the corneal reflection of an infrared light source. Stimuli were set and presented by Experiment Center software, and eye-movements were extracted using Begaze program. The stimuli were displayed on a 21inch LCD monitor.

**Figure 1:**
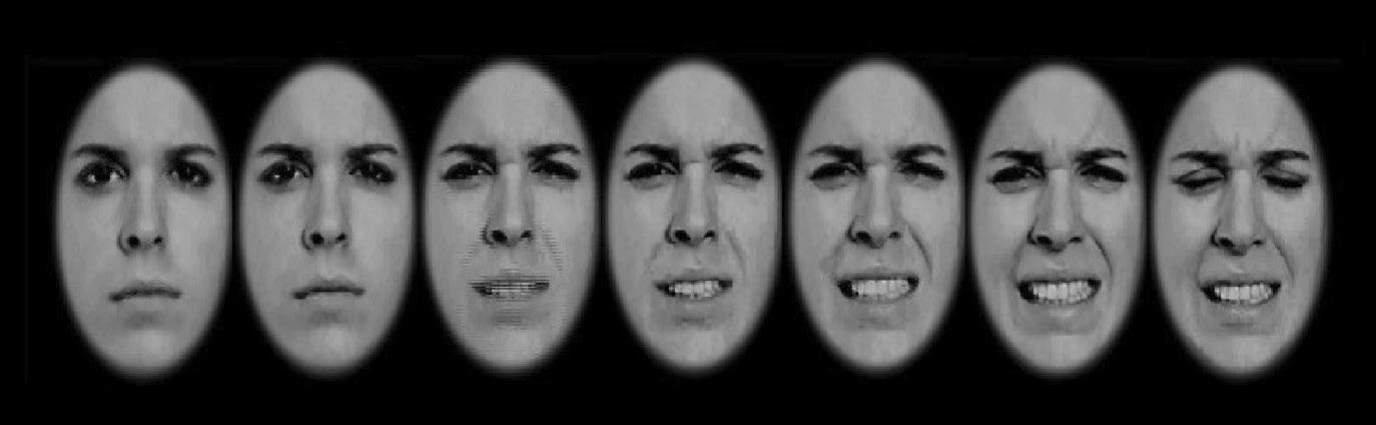
Process of change during the presentation of dynamic faces.

The facial expression stimuli were taken from the STOIC database^42^, which recruited actors to create the stimuli. These dynamic facial expressions were used in previous studies^43,44^. The dynamic faces used in the present study consisted of six adult faces (3 female) depicting pain, angry, happy, or neutral facial expressions. The original videos consisted of 15 frames and the rate of 30 Hz displaying. We increased the duration of each video to 800 ms and faces were resized to 60 mm × 74 mm in dimension (approximately 5.7 × 7.0 visual degree) using the Adobe Premiere Pro (Adobe Systems, 2015). Stimuli were grey scaled and their luminance and contrast were calibrated. All non-facial features (hair, ears, and neck) were removed using a mid-gray elliptical mask, and they were presented against a uniform black background (See Fig. 1).

### The antisaccade and prosaccade tasks^45^

The task was adapted by the antisaccade task, which is widely used in the anxiety literature^20^. Each trial began with a fixation cross (1.15° × 1.15°) that remained on the screen for 1,600 ms in the centre of the monitor. Participants were instructed to fixate on the cross until it disappeared. Then a single face appeared at 12.3 visual degree to the side of the fixation cross. In half the trials, the face appeared to the left and in the other half it appeared to the right. In the antisaccade task, participants were asked to look at the opposite side of the screen (i.e., the mirror position of the image), as quickly as possible without looking at the presented image. For the prosaccade trials, they were asked to look at the image as quickly as possible. The next trial was presented following an inter-trial interval, which was randomly scheduled to be between 700 ms and 1,300 to reduce the monotonous nature of the task^46,47^.

**Figure 2:**
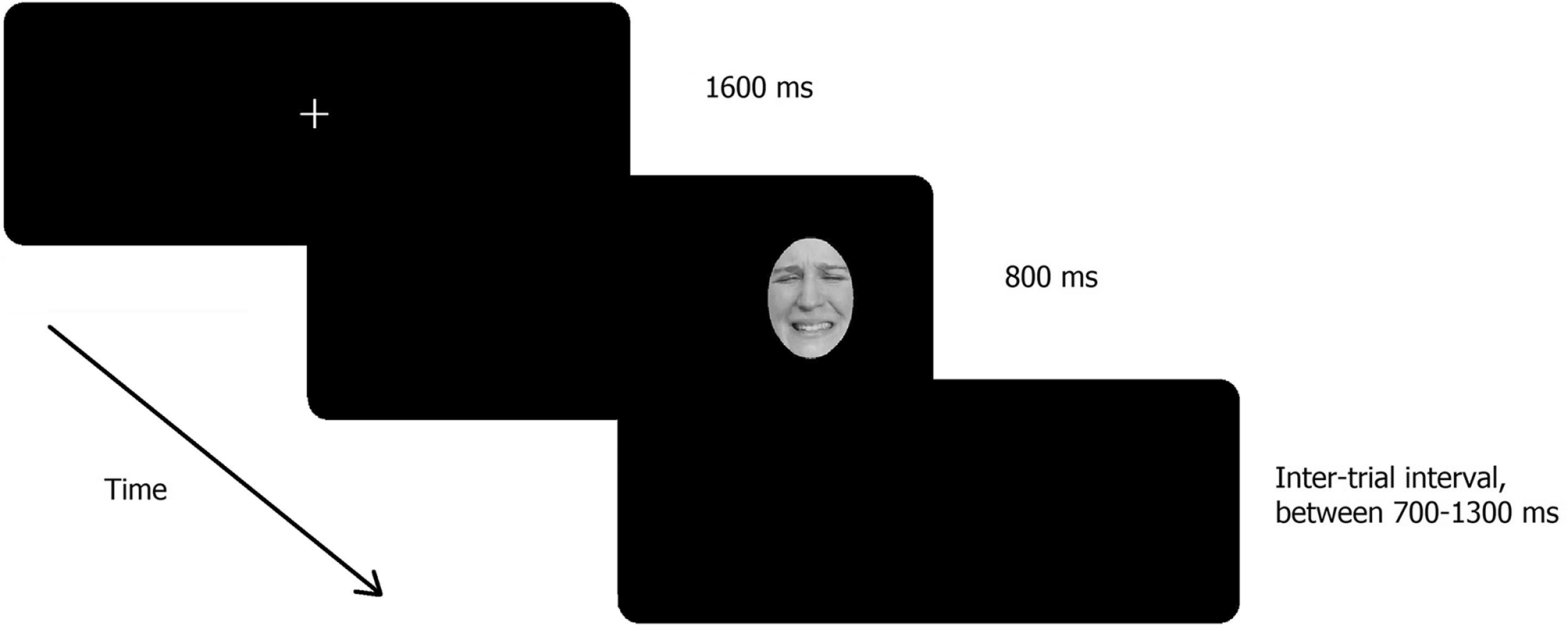
Example of a trial with a pain face.

A total of 192 trials were presented over two blocks, with one antisaccade block and one prosaccade block which were counterbalanced in order of presentation. Each block consisted of 96 trials, including 24 pain, 24 angry, 24 happy, and 24 neutral faces. Trials were counter-balanced both for gender and left–right presentation on the screen. The order of blocks was counterbalanced with half of the participants assigned to the antisaccade set first and the other half to prosaccade set first. The experimental paradigm is illustrated in Fig. 2.

### Procedure

At the beginning of the session, participants read the information sheet and gave informed consent. Participants were told that the aim of the study was to examine visual perception and accuracy of eye movements. Participants were then seated at a distance of 60 cm in front of a monitor in a sound-proofed room where they were assessed individually. Their chin was placed on a vertically adjustable chin rest to reduce head movements and increase the accuracy of eye tracking. Participants were informed that they would complete a computer task where they would see an image on the computer and that there would be two blocks of trials, with a short break in between each block.

A nine-point calibration procedure preceded ten practice trials prior to the commencement of the first block. At the beginning of each block, the instructions were presented on the screen and explained by the experimenter. Participants were asked to respond as quickly and accurately as possible. The main experimental task began after successful calibration (error less than 1 VA). After finishing the task, participants completed the questionnaires, were debriefed and thanked.

### Data preparation and analysis plan

Data were extracted using the BeGaze software (SensoMotoric Instruments GmbH., Teltow, Germany). To be defined as a saccade, eye movements needed to be made during the presentation of stimuli. In order to exclude anticipatory saccades, only saccades that occurred 83 ms or more after stimulus presentation were included in the analyses^48,49^. Similarly, only saccades that commenced from the centre of the screen (i.e. the fixation point) were included. The first saccade needed to be more than 3 degrees in amplitude and have a velocity threshold exceeding 30º/sec^46,50^. Ninety-two percent of all trials recorded saccades that met these criteria and were included in the analysis (the remaining 8% of trials were excluded). To assess task performance, we calculated two indices: (1) ***response accuracy*** that was calculated as the percentage of trials on which the participant made an error by dividing the number of trials with incorrect saccade responses by the number of trials for which a reliable saccade was recorded^49^, and (2) ***latency of first correct saccade*** that we indexed as the time between the onset of the stimuli and the initiation of the saccade in the correct direction. Furthermore, we calculated saccade latency bias scores, which we refer to as *attentional bias*, by subtracting the mean latency of prosaccade trials from antisaccade trials (the antisaccade cost) for each emotion and then subtracting the antisaccade cost for neutral faces from other emotions (pain, angry, happy)^27^. Larger saccade latency bias scores (attentional bias) show more difficulty in shifting attention away from emotional faces compared with neutral faces.

All statistical analyses were performed using SPSS (IBM Cop. V. 22). To examine the differences between groups, based on pain catastrophizing, in demographic characteristics and questionnaires data, X^2^ and t-tests were used for categorical and continuous variables respectively. To examine differences in saccade error rate and latency, a series of repeated measures Analyses of Variance (ANOVA) with trial type (antisaccade versus prosaccade) and valence (pain vs. angry vs. happy vs. neutral) as the within group factors and group (high versus low levels of pain catastrophizing) as the between group variable were performed. Where significant differences were found in ANOVAs, t-tests were used to further explain the effect(s). To control the false discovery rate in multiple testing, the Benjamini–Hochberg correction was used^51^. To quantify effect sizes of observed results, partial eta-squared *η*_*p*_^2^ and Cohen’s *d* were calculated (with 95% confidence intervals (CIs)). One problem that has plagued the attentional bias literature is the unreliability of the tasks used to assess attentional bias, such as the dot-probe^12^. Therefore, we conducted split half reliability for error rates and latency scores for each stimulus valence.

Moderator analyses were conducted using the Hayes and Preacher method^52^, and the PROCESS syntax^53^. PROCESS model 1 was applied which estimates a moderation model with a single moderator of the effect of an independent variable (pain catastrophizing) on a dependent variable (attentional bias to pain faces) by virtue of different levels of a moderating variable (attentional control scale scores). Interpretation of moderator effects was facilitated through a simple slopes analysis. Finally, we re-ran exploratory analyses using the two subscales of the ACS (i.e. focusing or shifting) to determine whether one or other aspect of attentional control might best account for the findings.

## Results

### Preliminary analyses

Participants with higher levels of PCS (*M* = 31.9, *SD* = 5.37) showed significantly greater trait anxiety, fear of pain, depression and lower ACS scores compared with those with lower PCS (*M* = 18.29, *SD* = 5.52) (see Table 1). The PCS scores were associated with a number of baseline characteristics including age (r = − 0.25, p = 0.008), depression (r = 0.23, p = 0.01), attentional control (r = − 0.24, p = 0.011). Women had higher catastrophizing scores than men (t = 2.16, p = 0.033). People who had higher levels of catastrophizing, also had higher levels of depression, as expected^54^. Similarly, differences in attention control were expected to be related to anxiety-related constructs (see Shi et al.^55^). Therefore, we did not see the need to control for these variables. We did, however, repeat our analyses controlling for depression, gender and age in the analyses, but since the pattern of results from the Analysis of Covariance (ANCOVA) was identical to those of the ANOVA, only the latter are reported here.

**Table 1:** Comparison between characteristics of two groups with high and low levels of pain catastrophizing.

		High PCS	Low PCS	t(108)	*p value*
Age		18.80 (0.89)	19.24 (1.98)	− 1.44	0.15
Years of education		13.25 (0.74)	13.27 (0.71)	− 0.12	0.91
State anxiety		39.92 (13.19)	37.66 (9.56)	1.01	0.31
Trait anxiety		43.84 (10.06)	39.66 (9.47)	2.24	0.03
Depression		15.86 (9.80)	10.49 (9.22)	2.96	0.004
Attentional control		48.78 (6.94)	52.25 (8.19)	− 2.37	0.019
Fear of pain	88.65 (18.18)		81.59 (18.03)	2.04	0.044

PCS, pain catastrophizing scale.

The importance of routinely reporting the reliability of behavioural measures has been emphasized recently^56^. The odd–even split half reliability was used to gain the internal consistency of the antisaccade errors and antisaccade/prosaccade latency scores across all participants for each facial type in the study. Split-half correlations were computed between odd and even items and the Spearman-Brown prophecy formula used to correct for test length. The split-half reliability of antisaccade errors was 0.85 for each emotion type and 0.96 across all trials. With respect to latency scores, the reliability scores were excellent for both antisaccade and prosaccade trials (0.95–0.98).

### Saccade error rates

Means and standard deviations of pro- and antisaccade error rates and latency for participants with high and low pain catastrophizing are shown in Table 2. For error rates, a series of repeated measure ANOVAs with trial type (antisaccade vs. prosaccade), and face emotions (pain vs. angry vs. happy vs. neutral) as the within subject variables and group (High PCS vs. Low PCS) as the between subject variable were conducted. The ANOVAs revealed a main effect of trial type [*F*(1, 108) = 118.27, p < 0.001; *η*_*p*_^2^ = 0. 52; 95% CI (0.41–0.6)], indicating higher error rates for antisaccade trials compared with prosaccade trials, as expected. The main effect of face emotions was significant as well [*F*(1, 108) = 4.41, p = 0.005; *η*_*p*_^2^ = 0.039; 95% CI (0.001–0.11)], indicating higher error rates for pain faces compared with angry [*t*(109) = 2.88, *p* = 0.005, Cohen’s *d* = 0.27; 95% CI (0.08–0.46)] and happy faces [*t*(109) = 2.77, *p* = 0.007, Cohen’s *d* = 0.26; 95% CI (0.07–0.45)]. This main effect was qualified by a two-way interaction between trial type and face emotion [*F*(1, 108) = 4.74, p < 0.003; *η*_*p*_^2^ = 0.042; 95% CI (0.002–0.12)], and the three-way interaction between group, trial type and face emotion interaction [*F*(1, 108) = 2.82, p = 0.039; *η*_*p*_^2^ = 0.025; 95% CI (0.001–0.09)]. No other main or interaction effect was found (all *F*s ≤ 1.92, *p*s > 0.13; *η*_*p*_^2^ ≤ 0.017).

**Table 2:** Means and standard deviations of antisaccade and prosaccade error rates and latency for participants with high and low pain catastrophizing.

Variable	Valence	High PCS		Low PCS	
Type		M	SD	M	SD
**Antisaccade**					
*Error rate (%)*	Pain	14.83	13.43	13.09	14.46
	Angry	9.72	9.95	12.46	13.71
	Happy	11.66	13.19	11.08	11.64
	Neutral	12.54	12.48	12.72	13.36
*Latency (ms)*	Pain	243.66	45.04	236.38	53.05
	Angry	242.07	44.84	236.46	51.57
	Happy	240.67	42.58	242.42	57.25
	Neutral	238.23	45.13	235.83	43.60
**Prosaccade**					
*Error rate (%)*	Pain	0.09	0.63	0.15	0.83
	Angry	0.48	1.78	0.14	0.76
	Happy	0.08	0.58	0.07	0.54
	Neutral	0.45	1.67	0.38	1.27
*Latency (ms)*	Pain	152.04	24.30	150.21	22.29
	Angry	145.81	23.48	146.77	22.25
	Happy	149.69	21.73	149.34	23.01
	Neutral	145.71	21.74	147.39	22.56

To understand the results more clearly, we conducted paired t-tests for face type (pain, angry, happy, and neutral) separately for prosaccade and antisaccade trials and for each PCS group. For prosaccade trials, the only significant difference observed was that amongst those low in PCS, there were more errors for happy than neutral faces: *t*(58) = − 2.09, *p* = 0.041, Cohen’s *d* = 0.27; 95% CI (0.01–0.53). No significant difference was found for those with higher PCS scores [all *t*(50) ≤ 1.5, *p*s > 0.14]. For antisaccade trials, there was no significant difference between faces for participants with low PCS, but those with high PCS showed higher error rates for pain faces than angry faces [*t*(50) = 4.05, *p* < 0.001, Cohen’s *d* = 0.57; 95% CI (0.27–0.86)] and happy faces [*t*(50) = 2.46, *p* = 0.017, Cohen’s *d* = 0.34; 95% CI (0.06–0.62)] but not neutral faces [*t*(50) = 1.99, *p* = 0.052]. There were also significant differences between angry and neutral faces [t(50) = − 2.32, p = 0.024, Cohen’s *d* = 0.32; 95% CI (0.04–0.6)].

### Saccade latency

For saccade latency, a 2 (group: high vs low PCS) × 2 (trial type: antisaccade vs. prosaccade) × 4 (face emotions: pain vs. angry vs. happy vs. neutral) ANOVA was performed. Consistent with the results for error rates, the results revealed a significant main effect for trial type [*F*(1, 108) = 501.67, p < 0.001; *η*_*p*_^2^ = 0.82; 95% CI (0.77–0.85)], indicating that prosaccades were faster compared with antisaccades. The main effect of face emotions was significant too [*F*(1, 108) = 4.18, p = 0.006; *η*_*p*_^2^ = 0.037; 95% CI (0.001–0.11)], indicating longer latency scores for pain faces compared with neutral [*t*(109) = 2.82, *p* = 0.006, Cohen’s *d* = 0.27; 95% CI (0.08–0.46)] and angry faces [*t*(109) = 2.37, *p* = 0.02, Cohen’s *d* = 0.22; 95% CI (0.04–0.41)], and longer latency score for happy faces compared with neutral faces [*t*(109) = 2.76, *p* = 0.007, Cohen’s *d* = 0.26; 95% CI (0.07–0.45)]. No other significant main or interaction effects were observed [all *F*s ≤ 1.51, *p*s > 0.21; *η*_*p*_^2^ ≤ 0.014].

### Moderation analyses

To test the hypothesis that attentional control moderates the association between pain catastrophizing and attentional bias for pain faces, a moderation model was tested with all participants. The dependent variable in this model was attentional bias to pain faces and the independent variable was PCS and the moderator variable was ACS scores. The moderation effect was significant (B = 0.075, 95% CI [0.011, 0.14], t = 2.31, p = 0.022).

Exploration of the conditional effect of PCS on attentional bias at values of the attentional control revealed the following:

1. When attentional control is low (one SD below the mean) there was no significant relationship between PCS and attentional bias to pain faces [*B* = − 0.305, 95% CI [− 1.03, 0.42], *t* = − 0.84, *p* = 0.40].
2. At the mean value of attentional control (within one SD of the mean), there was no relationship between PCS and attentional bias to pain faces [*B* = 0.224, 95% CI [− 0.35, 0.79], *t* = 0.77, *p* = 0.44].
3. At a high level of attentional control (one SD above the mean), there was a significant positive relationship between PCS and attentional bias to pain faces [*B* = 0.828, 95% CI [0.043, 1.61], *t* = 2.09, *p* = 0.039].

**Figure 3:**
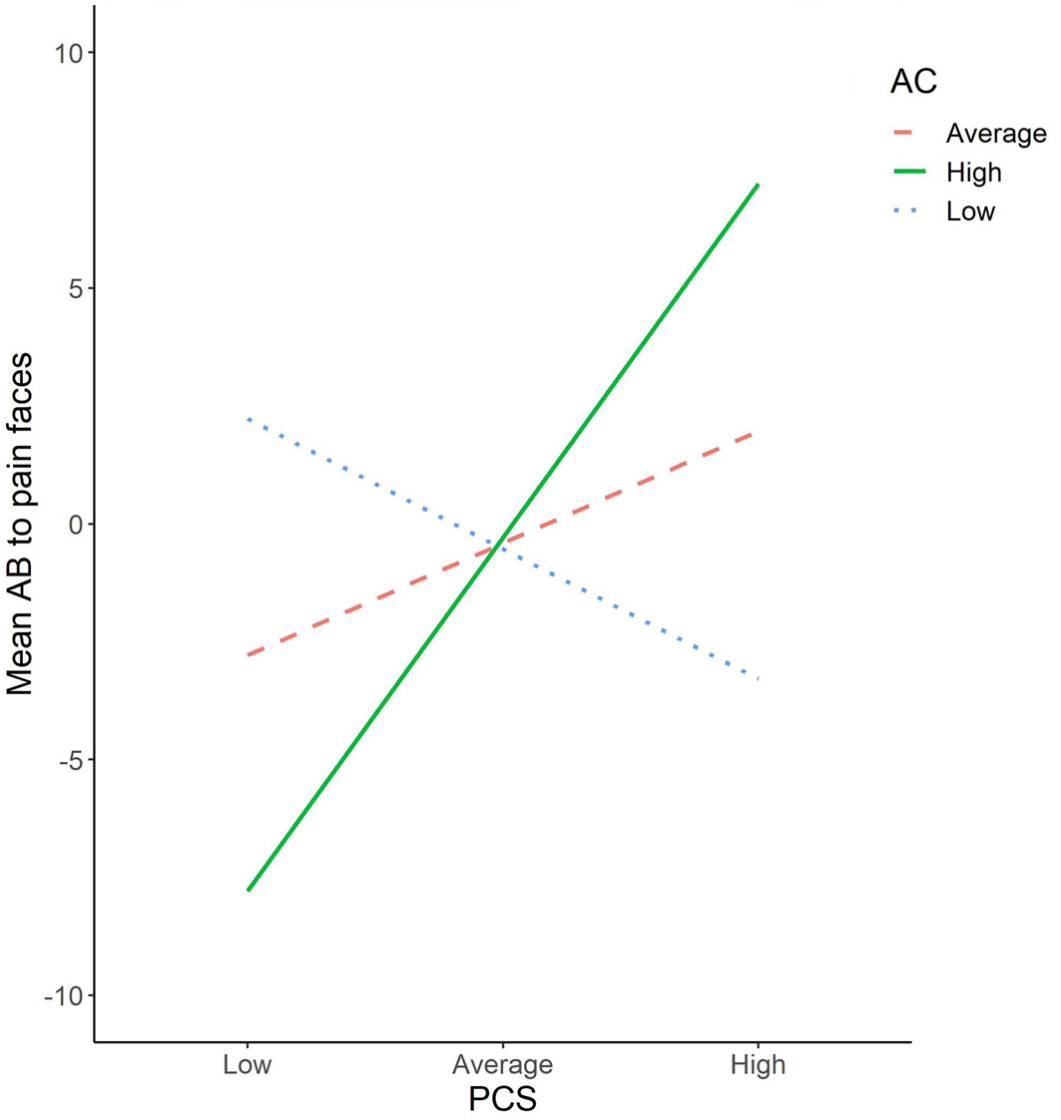
Simple slopes equations of attentional control (AC) moderation effect on pain catastrophizing and attentional bias to pain faces. Green = high AC, dashed red = average AC, dotted blue = Low AC.

Figure 3 demonstrates the conditional effects described above, confirming that there is a positive relationship between pain catastrophizing and attentional bias to pain faces, only at high levels of attentional control.

### Post-hoc analyses

In post-hoc analyses, we examined the two subscales of the ACS separately to determine whether the moderating role of attentional control was better accounting for by processes involved in focusing or shifting attention. Results showed that it was the shifting score that moderated the relationship between pain catastrophizing and attentional bias to pain faces (*B* = 0.20; 95% CI (0.70, 0.33); *t* = 3.53; *p* = 0.003). The pattern of the moderation was identical to that reported for the full scale. That is, at high levels of attentional control, the relationship between PCS and attentional bias was strong and positive, indicating that those who catastrophize more in relation to pain had more attentional bias towards pain. However, this relationship was absent at lower levels of attentional control. For the focusing subscale, there was no evidence of significant moderation.

## Discussion

The aim of this study was to determine whether people high in pain catastrophizing demonstrated attentional bias to pain-related faces in comparison to those low in pain catastrophizing using dynamic pain faces and the antisaccade task. Further, we aimed to determine whether these effects were moderated by the level of attentional control, such that the relationships were stronger in people with high attentional control. Our results confirmed that for error rates, participants high in pain catastrophizing were more likely to make errors in relation to painful faces in comparison to angry or happy faces, although the difference with neutral faces failed to reach significance. In contrast, the effect of pain catastrophizing on response latency bias in the antisaccade task was not significant. The latter null findings, however, were further clarified by the moderating role of attentional control. That is, the relationship between pain catastrophizing and attentional bias to pain faces was only significant in those people who reported a high level of attentional control.

These findings have important theoretical implications and may clarify why it has been so difficult to establish relationships between pain catastrophizing and attentional biases to pain. Fear-avoidance models of chronic pain suggest that the degree to which individuals pay attention to pain-related stimuli contributes to the vicious cycle in which they avoid pain-provoking tasks, become more disabled and experience increased pain. Pain catastrophizing is theorized to be one proximal factor that gives rise to ‘hypervigilance’^2,57^ but to date, relationships between pain catastrophizing and attentional bias have not been consistently found^7^. Our findings contribute to a small, but growing number of studies that show that attentional control may be the missing variable.

The results of the present study are consistent with studies by Heathcote and colleagues^16,17^. In their first study, they found that poorer attentional control was related to increased vigilance to pain faces only in adolescents with high pain catastrophizing. In their second study they found that among children with low attentional control, the higher their anxiety, the more they avoided looking at pain faces. In contrast, for those high in attentional control, the relationship was reversed. Our results are also consistent with the results of Lau et al.^18^. Lau et al. found that among youth with interfering pain from a community sample, attentional bias was observed, but only under conditions of low perceptual load. This is because under conditions of high attentional load, attentional control resources are unavailable to participants, indicating that attentional control is necessary for the relationship between attentional bias and pain to be observed.

In our study, there were differences amongst all those with high pain catastrophizing in the ability to inhibit attention towards pain-related faces (error rates) compared with happy and angry faces, but the speed with which all participants were able to inhibit their attention was impacted by pain catastrophizing only when attentional control was high. Furthermore, post-hoc analyses showed that this was not related to self-reported ability to focus attention, but rather the ability to shift attention. Only one recent study has investigated the moderating role of attentional control in adults with chronic pain between attentional bias and pain catastrophizing^19^. Mazidi et al.^19^ used a dot-probe task with eye-tracking (with 1500msec presentation). They found a general pattern of vigilance-avoidance amongst people with and without chronic pain and a moderation effect of attentional control in the relationship between pain catastrophizing and attention to happy faces. Specifically, for those patients with higher attentional control, those who also had higher pain catastrophizing attended more to happy faces. Furthermore, this study found, in contrast to the present results, that it was focusing (and not shifting) that moderated this effect.

It seems likely that the differences in the nature of the tasks might account for the different findings. Mazidi et al.^19^ used a dot-probe task and the moderation effect was found for sustained attention (i.e. overall dwell time) in people with chronic pain. Results suggested that it was the ability to focus attention that moderated the relationship between attention bias and pain catastrophizing. Results suggested that when people with chronic pain reported a high level of attentional focus, those with high levels of pain catastrophizing were more likely to focus overall on happy (not pain) faces compared with neutral faces, consistent with the overall pattern of vigilance-avoidance. In contrast, in this study, we used the antisaccade task, which assesses the ability to inhibit the initial attention towards pain faces. Only for those with high levels of attentional control was there a relationship between pain catastrophizing and a response latency (more difficulty inhibiting attention to the pain face). Essentially in the antisaccade task, it is the ability to shift attention from the distractor stimulus and inhibition of prepotent response, that is being assessed^25^, whereas for overall dwell time on the dot-probe it is arguably the ability to disengage or avoid the stimuli (see^58^). Therefore, it makes sense that shifting would be the aspect of attention important for the antisaccade task, whereas focusing would be more relevant to the task used by Mazidi et al.^19^. While this interpretation seems intuitive, it is important to note that the analyses of shifting versus focusing in both studies were post-hoc which is a limitation. Future research should systematically investigate which aspects of attentional control are relevant for which aspects of attentional bias.

Despite careful attention to methodology, there were limitations that should be borne in mind in interpreting the results. Firstly, the sample used in this study were healthy pain-free participants and therefore the degree to which the results would apply to people with chronic pain is unclear. Secondly, the sample in this study consisted mainly of women. There is evidence of the impact of gender on pain outcome^59,60^. Recruiting a more balanced sample would allow future researchers to examine gender-related factors and improve the generalizability of the results. Thirdly, the assessment of attentional control was made using self-report and is subject to the limitations of the self-report assessment^61,62^, however, the ACS covers a broader formulation of attentional control compared with a single behavioural task^62^. While the antisaccade task can be used to assess attentional control, it is the reaction time to neutral trials which is indicative of attentional control. Since this same variable was used to calculate the attentional bias scores, it would not have provided an independent measure of attentional control. Finally, it should be noted that the faces used in the present study were posed emotions by actors and do not represent spontaneous and authentic expressions. Studies show differential neural activity when judging posed versus genuine facial displays of emotions^63^. Future research can address how this might impact attentional processes to pain facial expressions. These limitations notwithstanding, the current study is the first to use the antisaccade task in relation to pain-related stimuli and used more ecologically valid stimuli with the inclusion of dynamic faces. The results have important implications.

Firstly, these results suggest that one reason why constructs such as pain catastrophizing have not been reliably associated with attentional biases in pain (e.g. Crombez et al.^7^ is that they are only associated amongst those with high levels of attentional control. The effect of pain on attention itself has long been described (see^4^). Although attentional control has rarely been studied in chronic pain samples, a recent meta-analysis has found that experimentally induced pain reliably affects some aspects of attention, notably orientation^64^. Further, there is evidence of small to moderate deficits in executive function (a construct related to attentional control) in people with chronic pain compared with those without^65^. These results suggest that further study of attentional control in chronic pain would be worthwhile, since in other areas of literature, such as anxiety research, deficits in attentional control have proved crucial in understanding of attention bias.

In the anxiety literature it is now established that deficits in attentional control are associated with greater severity of anxiety^55^, and a moderating role of attentional control has been found between attentional bias to threat and anxiety severity^66^. Further, in anxiety, attentional control has been found to moderate the efficacy for interventions that aim to modify biases, specifically Attention Bias Modification (ABM)^67^. ABM uses attentional bias paradigms (typically the dot-probe) to modify selective attention with a view to improving outcomes. Despite mixed results, ABM has been found to be efficacious in the prevention and treatment of anxiety (see^68^ for a review of meta-analyses). In chronic pain, however, despite early trials finding evidence that ABM improves pain-related outcomes (e.g.^69,70)^, others have failed to find an effect^71^. Importantly, none of the available trials have found the predicted change in attention bias, even when positive clinical outcomes were evident.

Attentional control could explain the success of ABM in the absence of changes in attentional bias. For example, if ABM proved to be effective only for those with high levels of attentional control, this might explain why it is in some contexts effective (such as with adults;^69,70^), but not with adolescents^71^. Alternatively, while ABM aims to change attentional bias, ABM has also been shown to improve attentional control^72^. Heeren et al.^73^ found that ABM training (regardless of whether the training was biased towards threat, away from threat or unbiased [i.e. the placebo condition]) resulted in improvements in attentional control. In Post-Traumatic Stress Disorder (PTSD) clinical trials have also shown that the placebo training is efficacious in reducing PTSD symptoms, leading the authors to rename the placebo ‘attention control training’^74^. While speculative, our results suggest that attentional control and its role in attentional bias and ABM should be the focus of future research if we are to better understand the role of attention bias in pain.

In summary, this study confirmed that high levels of pain catastrophizing were associated with increased difficulty in inhibiting a response to pain faces compared with neutral faces. When participants were able to inhibit the response towards painful faces, pain catastrophizing did not affect how quickly participants were able to do so overall. However, level of attentional control did moderate this relationship, such that the relationship between response latency and pain catastrophizing was only significant amongst those with the highest level of attentional control. These results underscore the importance of assessing attentional control in the study of attentional biases in pain.

## Data Availability

The data of the current study are available from the corresponding author on reasonable request.

## References

[1] Todd J (2015). Towards a new model of attentional biases in the development, maintenance, and management of pain. Pain.

[2] Vlaeyen JW, Linton SJ (2000). Fear-avoidance and its consequences in chronic musculoskeletal pain: a state of the art. Pain.

[3] Crombez G, Eccleston C, Van Damme S, Vlaeyen JWS, Karoly P (2012). Fear-avoidance model of chronic pain: the next generation. Clin. J. Pain.

[4] Eccleston C, Crombez G (1999). Pain demands attention: a cognitive-affective model of the interruptive function of pain. Psychol. Bull..

[5] Van Damme S, Crombez G, Eccleston C (2004). Disengagement from pain: the role of catastrophic thinking about pain. Pain.

[6] Eccleston C, Crombez G (2007). Worry and chronic pain: a misdirected problem solving model. Pain.

[7] Crombez G, Van Ryckeghem DML, Eccleston C, Van Damme S (2013). Attentional bias to pain-related information: a meta-analysis. Pain.

[8] Todd J, van Ryckeghem DML, Sharpe L, Crombez G (2018). Attentional bias to pain-related information: a meta-analysis of dot-probe studies. Health Psychol. Rev..

[9] Van Ryckeghem DML, Noel M, Sharpe L, Pincus T, Van Damme S (2019). Cognitive biases in pain: an integrated functional-contextual framework. Pain.

[10] Dear BF, Sharpe L, Nicholas MK, Refshauge K (2011). The psychometric properties of the dot-probe paradigm when used in pain-related attentional bias research. J. Pain.

[11] Skinner IW (2017). The reliability of eyetracking to assess attentional bias to threatening words in healthy individuals. Behav. Res. Methods.

[12] Dear BF, Sharpe L, Nicholas MK, Refshauge K (2011). Pain-related attentional biases: the importance of the personal relevance and ecological validity of stimuli. J. Pain.

[13] Khatibi A, Dehghani M, Sharpe L, Asmundson GJG, Pouretemad H (2009). Selective attention towards painful faces among chronic pain patients: evidence from a modified version of the dot-probe. Pain.

[14] Ceccarini F, Caudek C (2013). Anger superiority effect: The importance of dynamic emotional facial expressions. Vis. cogn..

[15] Sarapas C, Weinberg A, Langenecker SA, Shankman SA (2017). Relationships among attention networks and physiological responding to threat. Brain Cogn..

[16] Heathcote LC (2015). The relationship between adolescents ’ pain catastrophizing and attention bias to pain faces is moderated by attention control. Pain.

[17] Heathcote LC (2017). Child attention to pain and pain tolerance are dependent upon anxiety and attention control: an eye-tracking study. Eur. J. Pain.

[18] Lau JYF (2019). Greater response interference to pain faces under low perceptual load conditions in adolescents with impairing pain: a role for poor attention control mechanisms in pain disability?. J. Pain.

[19] Mazidi M (2019). Time-course of attentional bias to painful facial expressions and the moderating role of attentional control: an eye-tracking study. Br. J. Pain.

[20] Hutton SB, Ettinger U (2006). The antisaccade task as a research tool in psychopathology: a critical review. Psychophysiology.

[21] Magnusdottir BB (2019). Cognitive measures and performance on the antisaccade eye movement task. J. Cogn..

[22] Derakshan N, Eysenck MW (2009). Anxiety, processing efficiency, and cognitive performance new developments from attentional control theory. Eur. Psychol..

[23] Sweeney JA, Rosano C, Berman RA, Luna B (2001). Inhibitory control of attention declines more than working memory during normal aging. Neurobiol. Aging.

[24] Wieser MJ, Pauli P, Muhlberger A (2009). Probing the attentional control theory in social anxiety: an emotional saccade task. Cogn. Affect. Behav. Neurosci..

[25] Derakshan N, Ansari TL, Hansard M, Shoker L, Eysenck MW (2009). Anxiety, inhibition, efficiency, and effectiveness. An investigation using antisaccade task. Exp. Psychol..

[26] Dias NR (2015). Anti-saccade error rates as a measure of attentional bias in cocaine dependent subjects. Behav. Brain Res..

[27] Reinholdt-Dunne ML (2012). Anxiety and selective attention to angry faces: an antisaccade study. J. Cogn. Psychol..

[28] Kim M (2019). Dysfunctional attentional bias and inhibitory control during anti-saccade task in patients with internet gaming disorder: an eye tracking study. Prog. Neuropsychopharmacol. Biol. Psychiatry.

[29] MacLeod C, Mathews A, Tata P (1986). Attentional bias in emotional disorders. J. Abnorm. Psychol..

[30] Weierich MR, Treat TA, Hollingworth A (2008). Theories and measurement of visual attentional processing in anxiety. Cogn. Emot..

[31] Sullivan MJL, Bishop S, Pivik J (1996). The pain catastrophizing scale: development and validation. Psychol. Assess..

[32] Akbari F, Dehghani M, Khatibi A, Vervoort T (2016). Incorporating family function into chronic pain disability: the role of catastrophizing. Pain Res. Manag..

[33] Khatibi A, Schrooten MG, Vancleef LM, Vlaeyen JW (2014). An experimental examination of catastrophizing-related interpretation bias for ambiguous facial expressions of pain using an incidental learning task. Front Psychol..

[34] Derryberry D, Reed M (2002). Anxiety related attentional biases and their regulation by attentional control. J. Abnorm. Psychol..

[35] Abasi I, Mohammadkhani P, Pourshahbaz A, Dolatshahi B (2017). The psychometric properties of attentional control scale and its relationship with symptoms of anxiety and depression: a study on Iranian population. Iran. J. Psychiatry.

[36] McNeil DW, Rainwater AJ (1998). Development of the fear of pain questionnaire–III. J. Behav. Med..

[37] Roelofs J, Peters ML, Fassaert T, Vlaeyen JWS (2005). The role of fear of movement and injury in selective attentional processing in patients with chronic low back pain: a dot-probe evaluation. J. Pain.

[38] Mazidi M, Vig K, Ranjbar S, Ebrahimi M-R, Khatibi A (2019). Attentional bias and its temporal dynamics among war veterans suffering from chronic pain: investigating the contribution of post-traumatic stress symptoms. J. Anxiety Disord..

[39] Spielberger C, Gorsuch R, Lushene R, Vagg P, Jacobs G (1983). Manual for the State-Trait Anxiety Inventory.

[40] Beck AT, Sreer RA, Brown GK (1996). Manual for the Beck Depression Inventory–II.

[41] Ghassemzadeh H, Mojtabai R, Karamghadiri N, Ebrahimkhani N (2005). Psychometric properties of a Persian-language version of the Beck Depression Inventory-Second edition: BDI-II-PERSIAN. Depress. Anxiety.

[42] Roy S (2007). A dynamic facial expression database. J. Vis..

[43] Simon D (2008). Recognition and discrimination of prototypical dynamic expressions of pain and emotions. Pain.

[44] Khatibi A, Vachon-Presseau E, Schrooten M, Vlaeyen J, Rainville P (2014). Attention effects on vicarious modulation of nociception and pain. Pain.

[45] Hallett PE (1978). Primary and secondary saccades to goals defined by instructions. Vision Res..

[46] Derakshan N, Salt M, Koster EHW (2009). Attentional control in dysphoria: an investigation using the antisaccade task. Biol. Psychol..

[47] Garner M, Mogg K, Bradley BP (2006). Orienting and maintenance of gaze to facial expressions in social anxiety. J. Abnorm. Psychol..

[48] Myles O, Grafton B, Clarke P, MacLeod C (2019). GIVE me your attention: differentiating goal identification and goal execution components of the anti-saccade effect. PLoS ONE.

[49] Chen NTM, Clarke PJF, Watson TL, MacLeod C, Guastella AJ (2014). Biased saccadic responses to emotional stimuli in anxiety: an antisaccade study. PLoS ONE.

[50] Ansari TL, Derakshan N, Richards A (2008). Effects of anxiety on task switching: evidence from the mixed antisaccade task. Cogn. Affect. Behav. Neurosci..

[51] Benjamini Y, Hochberg Y (1995). Controlling the false discovery rate: a practical and powerful approach to multiple testing. J. R. Stat. Soc. Ser. B.

[52] Hayes AF, Preacher KJ (2014). Statistical mediation analysis with a multicategorical independent variable. Br. J. Math. Stat. Psychol..

[53] Hayes AF (2009). Beyond Baron and Kenny: statistical mediation analysis in the new millennium. Commun. Monogr..

[54] Quartana PJ, Campbell CM, Edwards RR (2009). Pain catastrophizing a critical review. Expert Rev. Neurother..

[55] Shi R, Sharpe L, Abbott M (2019). A meta-analysis of the relationship between anxiety and attentional control. Clin. Psychol. Rev..

[56] Parsons S, Kruijt A-W, Fox E (2019). Psychological science needs a standard practice of reporting the reliability of cognitive-behavioral measurements. Adv. Methods Pract. Psychol. Sci..

[57] Vlaeyen JWS, Crombez G, Linton SJ (2016). The fear-avoidance model of pain. Pain.

[58] Cisler JM, Koster EHW (2010). Mechanisms of attentional biases towards threat in the anxiety disorders: an integrative review. Clin. Psychol. Rev..

[59] Edwards R, Eccleston C, Keogh E (2017). Observer influences on pain: an experimental series examining same-sex and opposite-sex friends, strangers, and romantic partners. Pain.

[60] Keogh E, Cheng F, Wang S (2018). Exploring attentional biases towards facial expressions of pain in men and women. Eur. J. Pain.

[61] *The science of self-report: Implications for research and practice. The science of self-report: Implications for research and practice.* (Lawrence Erlbaum Associates Publishers, 2000).

[62] Quigley L, Wright CA, Dobson KS, Sears CR (2017). Measuring attentional control ability or beliefs? Evaluation of the factor structure and convergent validity of the attentional control scale. J. Psychopathol. Behav. Assess..

[63] McLellan TL, Wilcke JC, Johnston L, Watts R, Miles LK (2012). Sensitivity to posed and genuine displays of happiness and sadness: a fMRI study. Neurosci. Lett..

[64] Gong W, Fan L, Luo F (2019). Does experimentally induced pain affect attention? A meta-analytical review. J. Pain Res..

[65] Berryman C (2014). Do people with chronic pain have impaired executive function? A meta-analytical review. Clin. Psychol. Rev..

[66] Taylor CT, Cross K, Amir N (2016). Attentional control moderates the relationship between social anxiety symptoms and attentional disengagement from threatening information. J. Behav. Ther. Exp. Psychiatry.

[67] Basanovic J, Notebaert L, Grafton B, Hirsch CR, Clarke PJF (2017). Attentional control predicts change in bias in response to attentional bias modification. Behav. Res. Ther..

[68] Jones EB, Sharpe L (2017). Cognitive bias modification: a review of meta-analyses. J. Affect. Disord..

[69] Carleton NR, Richter AA, Asmundson GJG (2011). Attention modification in persons with fibromyalgia: a double blind, Randomized Clinical Trial. Cogn. Behav. Ther..

[70] Sharpe L (2012). Is there a potential role for attention bias modification in pain patients? Results of 2 randomised, controlled trials. Pain.

[71] Heathcote LC (2018). Attention bias modification training for adolescents with chronic pain: a randomized placebo-controlled trial. Pain.

[72] Chen NTM, Clarke PJF, Watson TL, MacLeod C, Guastella AJ (2015). Attentional bias modification facilitates attentional control mechanisms: evidence from eye tracking. Biol. Psychol..

[73] Heeren A, Mogoaşe C, McNally RJ, Schmitz A, Philippot P (2015). Does attention bias modification improve attentional control? A double-blind randomized experiment with individuals with social anxiety disorder. J. Anxiety Disord..

[74] Badura-Brack AS (2015). Effect of attention training on attention bias variability and PTSD symptoms: randomized controlled trials in Israeli and U.S. Combat Veterans. Am. J. Psychiatry.

